# Report of Cases Treated in the Men’s Medical Wards, Nos. 1 and 2, of the Philadelphia Hospital, Blockley

**Published:** 1839-03-09

**Authors:** W. W. Gerhard

**Affiliations:** Attending Physician


*Report of Cases treated in the Men's Medical Wards, Nos.* 1 *and* 2, *of the Philadelphia Hospital, Blockley.* By W. W. Gerhard, M. D., Attending Physician.
(Report furnished by Dr. Barnes, Resident Physician.)

| No. | Name. | Age. | Diagnosis. | Duration of treatment. | Result. | Local treatment. | General treatment. | Remarks. |
|---|---|---|---|---|---|---|---|---|
| 1 | W. B. | 38 | Aneurism of Aorta; thickening of semi-lunar and tricuspid valves. | 2 months. | Remaining. | Scarified cups, No. 1, q. d. for 14 days. | ℞. Pulv. Digitalis gr. ss.<br>Ext. Hyosciam gr. i., q. t. d. | The digitalis was only given when the dropsical effusion, which he was at times subject to, required it. Under these circumstances, it acted as a most powerful diuretic. |
| 2 | P. M. G. | 32 | Anasarca; no disease of heart; albuminous urine. | 2 weeks. | Remaining; convalescent. | Warm bath, q. s. die. | ℞. Infus. Eupator. f℥ij., q.q.h.<br>℞. Pulv. Ip. et Opii gr. x., nocte. | Profuse diaphoresis was induced on the fourth day, which continued for some time after the discontinuance of all treatment. All œdema disappeared three days after the commencement of diaphoresis. |
| 3 | P. M. | 28 | Anasarca; disease of heart; albuminous urine. | 18 days. | Remaining; convalescent. | Warm bath, q. t. d. | ℞. Potass. bitart ℈j.<br>Jalapœ gr. x.., s. s. q. d.<br>℞. Pulv. Doveri gr. iij., q. s. h. | The œdema decreased gradually from the fifth day, and had almost entirely disappeared on the tenth. Purging moderate; diaphoresis slight. |
| 4 | D. F. | 42 | Ascites. | 10 days. | Discharged; cured. | Warm bath, quaque nocte. | ℞. Pulv. Scillæ,<br>Hydrg. Chlor. mit. āā gr. i.<br>Pulv. Digitalis gr. ss., t. in d. | On the second day diuresis commenced, and on the fourth the patient became slightly ptyalized when the prescription was discontinued. Diuresis continued until the ninth day, when he was attacked with an external disease, for the treatment of which he was removed to the surgical ward. |
| 5 | D. R. | 46 | Arachnitis, slight. | 7 days. | Remaining; convalescent. | Sinapisms to feet and ankles; Hirund. American. No. XL. to occiput; Emp. Epis. to temples. | ℞. Mag. Sulp. ℥i., Statim Sumend.<br>℞. Mass. Hydrg. gr. j.<br>Tart. Antim. gr. $\frac{1}{8}$th, q. s. h. | All symptoms of cerebral disease had disappeared on the fifth day, when he complained of intense pain in the sciatic nerves. Granville's strongest liniment was applied over seat of pain, which relieved him almost instantaneously. |
| 6 | J. S. | 61 | Bronchitis, and chronic disease of the heart. | 2 days. | Remaining; relieved. | Scarified cups, No. vii., to thorax; sinapisms to præcordial region. | ℞. Spts. æth. S. comp. f℥i.<br>Lac. Assafœt. f℥v. M.<br>S. f℥ss., q. s. h. | Pain and dyspnœa somewhat relieved. |
| 7 | J. M. C. | 30 | Bronchitis. | 5 weeks. | Remaining. | Scarified cups, No. vi., to thorax, right side, posteriorly. | ℞. Rad. Seneg. contus. ℥i.<br>Rad. Sanguinar. ʒiij.<br>Aquæ Bullient Oj. M.<br>S. f℥ij., q. q. h. | The bronchitis has assumed a chronic form, and is now becoming complicated with tubercular depositions. |
| 8 | D. K. | 29 | Catarrh. | 12 days. | Discharged; cured. | Pediluv. sinapisms, q. s. nocte. | ℞. Infus. Senegæ f℥ij. q. q. h. | This patient was also placed on the lichen islandicus, as a dietetic. |
| 9 | E. M. | 36 | Chronic diarrhœa. | 1 month. | Remaining; relieved. | | ℞. Pulv. Opii gr. ss., q. q. h.<br>℞. Bals. Copaib. ♏.iv., q. s. h.<br>℞. Tr. Opii gtts. xxx., per enema, q. nocte. | This patient entered the ward in a very debilitated state. By confining him to a strictly farinaceous diet, by slight stimulations, and by the free use of opiates, the diarrhœa has been in a great measure checked. Bals. Copaib. proved useless. |

| | | | | | | | | |
|---|---|---|---|---|---|---|---|---|
| 10 | T. M. G. | 32 | Dysentery. | 2 weeks. | Discharged; cured. | Catap. Humuli, to abdomen. | ℞. Hydr. Chlor. mitis. gr. i. Pulv. Ip. et Opii gr. iv., q. q. h. ℞. Tr. Opii gtts. xxx., per enema, quaque sex t. hora. | This patient had been labouring under severe dysentery for several days before his entrance,—at which time he had about sixty stools in twenty-four hours. The disease yielded as soon as the mercurial had produced a very slight effect upon the gums. All treatment was discontinued on the seventh day. |
| 11 | W. E. | 71 | Ossification of valves of heart; cyrrhosis of liver; anasarca. | 7 weeks. | Remaining. | Sinapisms over præcordial region. | ℞. Pulv. Digitalis. Pulv. Scillæ āā gr. i., t. t. d. pro. re. nata. | The treatment in this case was merely palliative, and the digitalis was only resorted to when occasional exacerbations occurred. |
| 12 | T. C. | 36 | Intermittent fever. | 9 days. | Cured; discharged. | | ℞. Quiniæ Sulp. gr. ij., q. t. h. ℞. Infus. Quassia, ℥ij., q. s. h. | All three cases of intermittent fever were put on the same course of treatment. The youngest patient of the three had had this disease much longer than either of the others, and was under treatment for a much longer period. |
| 13 | D. M. | 19 | " " | 1 month. | Cured; discharged. | | | |
| 14 | T. F. | 50 | " " | 8 days. | Cured; discharged. | | | |
| 15 | G. D. | 49 | Gastritis. | 11 days. | Discharged; cured. | Scarified cups No. viij. over epigastrium, followed by Catap. Humuli over abdomen. | ℞. Hydrg. Chlor. mitis. gr. ss. Pulv. Ip. et Opii gr. iv. M. q. t. h. | On the fourth day the gums became slightly affected, after which the patient recovered rapidly. |
| 16 | W. N. | 40 | Gastritis. | 3 days. | Discharged; cured. | Catap. Humuli over abdomen. | ℞. Mist. Oleag. f℥iij., q. s. h., until purged. | In this and the two following cases the gastritis was very slight, and yielded almost immediately after the operation of the purgatives. |
| 17 | G. D. | 58 | " | 2 days. | Discharged; cured. | Catap. Humuli over abdomen. | ℞. Hydg. C. mitis. gr. x. Pulv. Rhei ℈i., s. s. | |
| 18 | C. G. | 28 | " | 2 days. | Discharged; cured. | Dry cups No. viii. over Epigastrium. | ℞. Ol. Ricini f℥i. Tr. Opii gtts. x., s. s. | |
| 19 | J. L. | 35 | Lumbago. | 5 weeks. | Discharged; relieved. | Granville's strong liniment applied over lumbar region, q. s. d. | ℞. Pulv. Camphoræ gr. v. Pulv. Resin Guaiac. gr. xv., q. q. h. | The application of Granville's liniment produced a decided improvement in a few days. |
| 20 | J. M. | 32 | Lumbago. | 5 weeks. | Discharged; cured. | Scarified cups No. iv. to lumbar region, q. q. d.; Emp. Epis. over sciatic nerves. | ℞. Pulv. Ip. et Opii gr. iv., q. q. h. | This was quite a recent case, in which the pain upon motion was very severe. Moderate diaphoresis was produced and kept up by the P. Doveri. |
| 21 | T. P. | 49 | Neuralgia and hypochondriasis. | 3 weeks. | Remaining. | Scarified cups, No. vi., over upper dorsal vertebræ; Emp. Epis., 4 × 4, over lower part of sternum. | ℞. Mass. Hydrg. gr. iij., q. nocte sumend. ℞. Ext. Colocynth. gr. vi., q. mane sumend. | No improvement has as yet been noticed. The course of purgatives was commenced two days since. The neuralgic affection is chronic, with periodical exacerbations. |

| No. | Name. | Age. | Diagnosis. | Duration of treatment. | Result. | Local treatment. | General treatment. | Remarks. |
|---|---|---|---|---|---|---|---|---|
| 22 | T. K. | 28 | Pneumonia, with pericarditis. | 43 days. | Discharged; cured. | Scarified cups, No. viii., to thorax, rt. side, posteriorly; Emp. Epis., 4 × 4, over præcordial region. | ℞. Rad. Senegæ ℥i.<br>Rad. Sanguinar. ʒiij.<br>Aq. Bullient Oj., f℥jj., q. q. h. | The infusion acted as an expectorant, diaphoretic, and laxative. The pneumonia was cured in nine days,—the pericarditis remaining in a slight degree until the twentieth day from the commencement. |
| 23 | P. W. | 28 | Pneumonia, second attack; cured of the first and discharged ten days before, went to work, and on exposure the second recurred. | 3 days. | Dead. | Sinapisms to feet; Emp. Epis., 6 × 8, to right side of thorax; cups to right side after reaction. | ℞. Ammon. Carb. ʒi.<br>Spts. æth. S. comp. f. ℥ss.<br>Mucil. Acac. q. ft. f. ℥vi.<br>S. f. ℥ss. q. ss. h.<br>℞. Pulv. Dover, gr. iii.<br>Nitrat. Potass. gr. iv. twice only. | This patient was largely bled previous to entrance, and was brought to the hospital in a state of collapse, from slipping of bandage on the way. Reaction was fully established on the evening of the second day. Cerebral symptoms developed on second night, which increased in intensity until the time of his death. |
| 24 | J. A. | 32 | Pleuritis (chronic.) | 11 days. | Discharged; cured. | Emp. Epis., 4 × 6, to right side of thorax, kept open with Cerat. Resinæ for five days. | ℞. Mass. Hydrg. grs. ii. T. in die. | The patient complained on his entrance of pain in the right side of thorax; more especially upon a full inspiration. This pain was much relieved by the blister, and was entirely removed before the mercurial had produced ptyalism |
| 25 | W. C. | 28 | Pleuropneumonia. | 16 days. | Remaining; convalescent. | Emp. Epis., 6 × 8, to post. portion of thorax. Pediluv. Sinap. q. nocte. | ℞. Rad. Seneg. ℥ss.<br>Rad. Sanguinar. ʒiss.<br>Aquæ Bullient Oj.<br>f ℥ii. q. sex. h.<br>℞. Pulv. Doveri grs. iii. q. q. h. | The Pleuropneumonia is entirely cured, and the patient remains in the ward, merely on account of the debility ensuing from the severe attack under which he has laboured. |
| 26 | J. K. | 28 | Paraplegia. | 10 days. | Discharged relieved.; | Cucurb. cruent. No. iv., to spine, q. s. d. | ℞ Ext. Colocynth comp. grs. vi. q. secund. die. | This patient has been transferred to another ward, where he still remains under the same course of treatment, and is convalescing rapidly. |
| 27 | J. S. | 62 | Paraplegia of three years' duration. | 20 days. | Discharged. | Granville's mild liniment to spine, t. in die. | ℞. Pill, laxat. comp. No. iv. q. s. d. | Discharged to the incurable ward. |
| 28 | J. B. | 65 | Paraplegia. | 23 days. | Discharged; convalescent. | Cucurb. cruent. No. iv,, to spine, q. s. d. | ℞. Infus. Sennæ f. ℥iv. q. s. d.<br>℞. Venesect f. ℥xvi. | Was discharged at his own request; the paraplegia being so much relieved that he could return to his occupation. |
| 29 | S. K. | 29 | Ptyalism. | 5 days. | Discharged; cured. | Catap. Humuli to throat. | ℞. Aquæ Opii f. ℥vi. } mouth<br>Pulv. Alumin. ʒi. } wash. | Convalescent from dysentery. |
| 30 | J. H. | 55 | Rheumatism. | 1 month. | Discharged; cured. | Emp. Epis. to knee; entire rest. | ℞. Pulv. Doveri grs. iii. q. in die. | The Rheumatism was confined to the right knee. |

| | | | | | | | | |
|---|---|---|---|---|---|---|---|---|
| 31 | R. F. | 50 | Rheumatism. | 2 months. | Discharged; cured. | Emp. saponis to ankle, covered with a tight bandage. | ℞. P. Resin Guaiac. gr. x., q. sex h. | The Rheumatism was confined principally to the foot and ankle. |
| 32 | J. D. | 32 | Acute Rheumatism and pericarditis. | 17 days. | Discharged; cured. | Cucurb. cruent. No. viii. over præcordial region. | ℞. Pulv. Rad. Colch. gr. vi. Pulv. Aromat. gr. iij., q.q.h. ℞. Decoc. Zinziberis Oj., in die. | The colchicum was taken every four hours for two days, every six hours for three days, and twice daily for two days. On the fourth day, all pain was removed, gentle diaphoresis established, and from that period, convalescence was gradual. Slight nausea the first day. |
| 33 | H. J. | 53 | Ramollisement of brain and third stage of Pneumonia. | 5 days. | Dead. | Sinapisms to feet; Emp. Epis. to nucha; Emp. Epispas. to thorax. | ℞. Ammon. Carb. ʒi. Syrup Senegæ f℥i. Mucil. Acac. gr. ft. f℥vi. M. S. f℥ss., q. h. Wine. | This patient's constitution had been much impaired by intemperate habits, and by a secondary syphilitic affection of several years' duration. He entered the medical wards in a state of asthenia. |
| 34 | J. M. | 45 | Spinal irritation. | 5 weeks. | Remaining; convalescent. | Granville's vesicating liniment to spine, q. s. d. | ℞. Pill. Cathart. Comp. No. iij., quaque die, secund. | The improvement in this case, since the application of Granville's liniment has been very marked. |
| 35 | I. L. | 35 | Phthisis pulmonalis. | 14 weeks. | Discharged. | | ℞. Syrup Senegæ. Syrup Tolu āā f℥i. Mucil. Acac. gr. ft. f℥vi., S. ℥ss. p. r. n. | Discharged to incurable ward. |
| 36 | J. D. | 45 | " " | 2 months. | Remaining. | | ℞. Ext. Hyosciami gr. iij. Pulv. Camphoræ gr. v., ft. pill, nocte sumend. ℞. Infus. Prunus. Virgin. Oj. in die. | This patient has a cavity in the upper lobe of right lung, which has not increased in size for the last month. No hectic, and no night sweats. |
| 37 | J. R. | 24 | " " | 7 weeks. | Remaining. | | Tonic infusions, demulcents, and expectorants, pro re nata. | Rapid softening of tubercles, accompanied with emaciation, prostration, hectic, &c. |
| 38 | T. R. | 22 | " " | 3 months. | Remaining. | Emp. Epispas. 2 × 2, to right axillary region, q. s. d. | ℞. Infus. Prunus. Virg. Oj. Acid. Sulp. Aromat. f℥ss., s. q. d. | Entered the hospital three months since with chronic pleuritis, which has been succeeded by tubercular depositions. Emaciation slight, cough severe, no hectic. |
| 39 | M. G. | 45 | " " | 6 weeks. | Remaining. | | ℞. Ext. Hyosc. gr. vi. Acet. f℥i Syrup Simp. f℥i. Mucil. Acac. q. ft. f℥vi. M. } f℥ss. q.s.h. | Cough severe, especially at night; emaciation gradual; slight hectic. |
| 40 | F. B. | 48 | " " | 3 weeks. | Remaining. | Emp. Epispas to left axillary region, kept open with cerat. resinæ. | ℞. Syrup Tolu, " Senegæ āā f℥i. Mucil. Acac. q. ft. f℥vi. M. ℥ss. q. s. h. | Has less cough and pain, and sleeps much better than at time of admission. No emaciation; no hectic. |

These tables include all the patients treated in two wards of the Philadelphia Hospital, in the time included between the dates which are specified. The diseases were those most frequent during the winter months of the present year. They include several points of interest, but are deficient from the accidental omission of the date of the disease at which the patients entered the hospital. In other respects, they have been prepared with great accuracy by Dr. Barnes.

The fatal cases were two in number; both from pneumonia, more or less complicated. One of these cases was that of a patient who had recovered from a previous attack of severe pneumonia, and had returned to his work a week before his entrance. He carelessly exposed himself to a heavy rain, and was taken with a return of the disease. The patient was bled in the city; on his way to the hospital the bandage became loose, and he lost a very large quantity of blood. At his entrance, he was in a state of perfect collapse; reaction was established with great difficulty; the patient afterwards improved, but at last died of cerebral symptoms. No autopsy could be made; but the patient, in all probability, died of inflammation of the brain, which had proved fatal to several patients affected with pneumonia in the early part of the winter. The previous attack of pneumonia had commenced with hæmoptysis, and the patient had been for some months in feeble health. The disease was, therefore, probably complicated with phthisis.

The second case was one of asthenic pneumonia. The patient entered in the third stage of the disease, and but few means of treatment were applicable. The sputa assumed the peculiar tinge which is usually met with in the last stage of pneumonia; that is, they were thin, and of a deep brown tint, similar to that of the juice of stewed prunes.

The pathological appearances were interesting rather on account of the lesions found in the brain, than those in the lungs. The left lung was hepatized throughout, and the inflammation was just passing from the second into the third stage. The brain offered a remarkable depression on the right hemisphere, near the upper termination of the fissure of Sylvius; the arachnoid adhered closely to the pia mater, and both these membranes were attached to a mass of cellular substance, which had replaced the ordinary cerebral matter, but had in a great measure retained its peculiar form. The convolutions were quite evident, were formed of the cellular substance, but were much thinner than usual. In a small part of the cellular mass, which was examined by the microscope, a portion of cerebral substance appeared to be inclosed in the meshes of the cellular tissue. The depth of the cellular substance was about an inch, and its breadth at least an inch and a half; it was surrounded by a portion of the brain, softened and of a yellowish tint. I regarded the lesion as an instance of the developement of the cellular substance of the brain; but, it was doubtful whether the natural cellular tissue which is supposed to exist in the brain, was merely developed in consequence of some previous inflammation, or whether the disease was simply the remains of an apoplectic clot. The previous history of the patient could not be ascertained, and at the time of his entrance his mind was too much enfeebled for him to give a correct account of his symptoms.

The treatment was as free from complications as was possible under the circumstances, and in all cases, the remedies were withheld as soon as their peculiar effect was produced. By taking this precaution, we were enabled greatly to diminish the quantity of medicine which was required, and to relieve the patient with the least possible disturbance of the alimentary canal. When diuretics or diaphoretics were administered, the discharge of urine and perspiration nearly always continued until the patient was freed from the effusion, and the mode of practice to which we adhered was therefore remarkably efficient. With other remedies, it was more difficult to apportion the dose, and to restrict the treatment to the minimum quantity; but in most cases of chronic disease, we may attain the results at which we aim, by giving smaller doses of medicine than are usually administered.

A new remedy, that is, new in the form in which it is now used, was prescribed in many cases of neuralgic disease: I allude to the ammoniacal liniment, the composition of which was first made known in this country, by its publication in the Medical Examiner, from a formula given by Dr. Granville, in the London Lancet. Notwithstanding the absurd charlatanism with which this particular form of ammoniacal liniment was recommended to the public, we were not disposed to forego the real advantages which might be derived from its application. It was applied in many forms of disease, in most cases adopting the precise formula of Dr. Granville, but in some, varying the proportions, or altogether omitting some of the ingredients; always retaining, however, the most essential, the water of ammonia. In many cases of neuralgic pains, the relief was immediate and permanent. In thoracic diseases, when it was desirable to keep up a long continued drain from a small surface, much advantage seemed also to result from those blisters, which did not at first give rise to as severe pain as a fly blister, while they continued open for a much longer period. The disadvantage of these applications arises from the slowness with which the vesicated surface heals, and its tendency to take on superficial ulceration; in neuralgic diseases, these are serious evils and they not unfrequently follow the application even of the milder liniment; but in cases where it is desirable to keep up a long-continued discharge, the ammoniacal irritants are certainly of great convenience and advantage. It is in these cases that Gondret's ointment, composed of lard and aq. ammoniæ, has been most employed. Whether any great diversity of action is observable in the various kinds of ammoniacal applications, is yet to be determined. On a future occasion, we hope to furnish sufficient facts to resolve this question, and ascertain whether the ammoniacal preparations are, in some cases, superior in power to other modes of counter-irritation.

